# Supplementary material for: Sociodemographic intersections and risk of multiple long-term conditions: A systematic review
Source: PLoS One. 2025 Nov 17;20(11):e0336958. doi: 10.1371/journal.pone.0336958 (PMC12622788; doi:10.1371/journal.pone.0336958)
Supplement: S2 Table — (DOCX) [file pone.0336958.s002.docx]

**S2 Table. Newcastle-Ottawa Scale risk of bias of reviewed studies**

| **Study** | [**Moreno-Juste et al. (2023)**](https://pmc.ncbi.nlm.nih.gov/articles/PMC9893716/) | [**Moreno-Juste et al. (2024)**](https://jogh.org/2024/jogh-14-04229) | [**Zandam, Akobirshoev,  and Mitra (2024)**](https://pmc.ncbi.nlm.nih.gov/articles/PMC10979663/) | [**Lam et al. (2024)**](https://www.tandfonline.com/doi/full/10.1080/00324728.2024.2331447#d1e158) |
| --- | --- | --- | --- | --- |
| **Selection** | | | | |
| Adequacy of the MLTC definition | Yes (★): with independent validation (directly from electronic health records) | Yes (★): with independent validation (directly from electronic health records) | Yes (★): with independent validation (directly from electronic health records) | Yes (★): with independent validation (directly from  waves of national surveys) |
| Representative of the cases | Yes (★): whole coverage of those with at least one long-term condition in Aragon, Spain from the national electronic health records system | Yes (★): whole coverage of those with MLTC (at least 2 or more long-term conditions) in Aragon, Spain from the national electronic health records system | Yes (★): Healthcare Cost and Utilisation Project Nationwide Emergency Department Sample (20%) | Yes (★): nationally representative household panel survey datasets |
| Selection of controls* | Community controls (★) | Community controls (★) | Hospital controls (☆) | Community controls (★) |
| Definition of controls* | Native, urban-dwelling,  middle-income males (★) | Native, urban-dwelling,  middle-high income females (★) | Non-disabled White individuals (★) | Those with less-than-secondary education in all ethnicities (★) |
| **Comparability** | | | | |
| Comparability based on the design or analysis | Controls for income, residence area, migrant status (★)  Controls for intersections of sociodemographic factors (★) | Controls for income, residence area, migrant status (★)  Controls for intersections of sociodemographic factors (★) | Controls for ethnicity and disability (★)  Controls for intersections of ethnicity and disability (★) | Controls for ethnicity and education (★)  Controls for intersections of ethnicity and education (★) |
| **Exposure** | | | | |
| Ascertainment of exposure | Secure record: national electronic health records (★) | Secure record: national electronic health records (★) | Secure record: Nationwide Emergency Department Sample (20%) (★) | Structured interview blind to case/control status (★) |
| Same method of ascertainment for cases and controls | Yes (★) | Yes (★) | Yes (★) | Yes (★) |
| Non-response rate | Same rate for both groups: whole-of-population electronic health records (★) | Same rate for both groups: whole-of-population electronic health records (★) | Same rate for both groups: nationwide 20% sample electronic health records for emergency department visits, hospitalisation, and deaths (★) | Non-responders described (☆) |
