# Supplementary material for: Sociodemographic intersections and risk of multiple long-term conditions: A systematic review
Source: PLoS One. 2025 Nov 17;20(11):e0336958. doi: 10.1371/journal.pone.0336958 (PMC12622788; doi:10.1371/journal.pone.0336958)
Supplement: Attachment — Submitted filename: PONE-D-25-21560_Response to Reviewers_17Sep2025.docx [file pone.0336958.s005.docx]

Mansuk Daniel Han

Leicester Real World Evidence Diabetes Research Centre

University of Leicester

United Kingdom

Email: mdh26@leicester.ac.uk

**Rebuttal Letter –** Manuscript Title: “Sociodemographic intersections and risk of multiple long-term conditions: a systematic review” (PONE-D-25-21560)

Dear PLOS One Editorial Team:

Thank you for your review of our manuscript, originally submitted as [PONE-D-25-21560] titled “Sociodemographic intersections and risk of multiple long-term conditions: a systematic review”. We appreciate the feedback we received.

Our response below corresponds to each point raised by the academic editor and reviewer #1, and we amended the manuscript accordingly. It should be noted that no changes have been suggested by reviewer #2, who congratulated us for the well-conducted study.

We are grateful for having the opportunity to revise and resubmit this work. We hope that the changes made will now meet the editorial team’s expectations.

Yours sincerely,

Mansuk Daniel Han on behalf of the authors.

**1. Abstract: Please clarify that only four studies were included, and emphasize the limitations upfront to better align expectations for the reader.**

- Revised abstract now includes: “Four studies met inclusion criteria but none were longitudinal, which limits our ability to examine the role of intersectional effects on MLTC outcomes over the life course from this review. A narrative synthesis was conducted due to their wide methodological heterogeneity. The limited evidence may potentially suggest that MLTC outcomes can vary considerably at the intersections of sociodemographic factors.”

**2. Introduction: The concept of intersectionality is introduced, but the theoretical framework requires further development. I suggest incorporating key foundational references (e.g., Crenshaw, Bowleg) and clarifying how intersectionality differs from statistical interaction or additive models.**

- Agreed; reworked and expanded
- Delved back into the literature on intersectionality theory [1, 2], quantitative application of intersectionality in public health [3-7], and critique on the social determinants of health [8, 9]

**The objective of the review should be more explicitly stated at the end of the introduction. Consider adding a sentence such as: “The aim of this systematic review is to…”**

- Revised: The aim of this systematic review is to examine how the interplay of sociodemographic factors plays a role on MLTC outcomes by their relational context. We identify and summarise quantitative studies that examine MLTC outcomes through an intersectionality lens; assess which social positions (e.g. gender, ethnicity, income) have been studied in combination; and discuss how their interplay in MLTC outcomes varies when considered within an intersectional context.

**3. Methods: The eligibility criteria are well defined; however, the rationale for excluding studies with specific populations (e.g., people with diabetes) could be better justified.**

- Removed the criterion upon further review.
- Review results remain the same: In retrospect what we actually had done was not restricted to the ‘general population’. For example, two of the four studies included in the final synthesis examine populations with at least one long-term condition [10] or already with multimorbidity [11].
- Theoretical justification: Current literature reveal that there is no rationale to restrict ourselves to a ‘general’ condition, because one of the key components of an MLTC trajectory includes the type of index condition [12-17].

**The description of how intersectional approaches were evaluated in the included studies lacks clarity. Please elaborate on the criteria used to determine whether studies “correctly” applied intersectionality analyses.**

- Revised paragraph: Particular attention was paid to whether the studies applied intersectionality analysis approaches correctly. Because the use of intersectionality approach in quantitative health research is in nascent stages [6, 18, 19], the claims of applying intersectionality in quantitative health research warrant case-by-case examination. Therefore, we examined each study by the criteria for improving quantitative intersectional research suggested by Bauer and colleagues [7].
- Revised the ‘study selection’ subsection: Upon further examination, one record [20] was also removed by following the criteria from by Bauer and colleagues [7]. The study invokes intersectionality but draws a conventional category-by-category comparison where contrasts are made by comparing within poverty levels and within ethnic groups separately.

**The use of the PICO framework is unconventional for this type of review. The term “control” as “unidimensional factors” is confusing and may not be necessary.**

- Agreed; removed Table 1 and associated text.

**4. Results: The presentation of the four included studies is informative, and the tables are helpful. However, given the very limited number of included studies — with two from the same cohort — the generalizability of findings should be presented more cautiously. In several parts of the text, findings are overstated given the small and heterogeneous evidence base. I recommend using more qualified language such as “may suggest” or “indicate a potential trend.”**

- Agreed, softened the language with more nuances where applicable.

**5. Discussion: The discussion offers a useful synthesis but occasionally draws strong conclusions from limited evidence.**

- Agreed, softened the language with more nuances where applicable.

**Please be more explicit about the implications of relying primarily on cross-sectional data and modeled projections.**

- Revised 1^st^ paragraph: MLTC builds along the cumulative disadvantages experienced across the life course [21, 22], starting with adverse childhood experiences and socioeconomic conditions [23, 24]. However this review includes three cross-sectional studies [10, 11, 25], two of which [10, 11] originated from the same cohort, and the fourth study was based on modelled projections [22]. It limits our ability to examine the effect of intersectional effects on MLTC outcomes over the life course from this review, for example the age of index condition onset [26-29]; temporal order of MLTC accumulation [14-17]; transition probabilities between MLTC states [16, 30, 31]; or speed of MLTC accumulation [29]. The limited evidence in this review, however, may still suggest that MLTC outcomes can vary considerably at the intersections of sociodemographic factors.

**The section on “solution-linked variables” is important but underdeveloped. Consider expanding this discussion to provide concrete examples of variables that could be used in future research or interventions.**

- Revised: Another key limitation of the existing evidence is the lack of solution-linked variables. Based on the original definition [32], solution-linked variables are variables which can be modified or intervened upon to mitigate intersectional inequalities in MLTC trajectories. However, like many other intersectionality-focused quantitative studies [33], the studies included in this review do not include such variables. Continuing to analyse inequalities in increasingly finer, intersectional details without suggesting actionable solutions may strengthen ideas that health inequalities are inherently natural among groups [7].

**The section on the morbidity-mortality paradox is interesting but could be shortened or more tightly linked to the intersectional focus of the study.**

- Agreed, we cut out the superfluous bits in the middle.
- Revised: The morbidity-mortality paradox, where “females live longer than males but spend a higher proportion of their total life expectancy in poorer health states” [34], was partially observed in three [10, 11, 22] studies where MLTC outcome disparities were examined by sex. However, it remains challenging to postulate whether these findings are generalisable without a deeper exploration of intersectional contexts of each study and further evidence besides the limited findings of this review.

**6. Limitations: I appreciate your candid acknowledgment of limitations. However, the limitation regarding publication language bias could be removed or softened unless you found evidence that it affected your results**

- No evidence that it affected the results; therefore removed.

**The limitation that three of the four studies are cross-sectional should be emphasized more strongly in relation to the study’s objectives.**

- See the revised 1^st^ paragraph of Discussion above.

**7. Conclusion: The conclusion accurately summarizes the study but could be strengthened by clearly stating what future research should prioritize (e.g., longitudinal data, use of solution-linked variables, inclusion of diverse populations, theory-informed methods)**

- Revised: This systematic review highlights the importance of applying an intersectional framework to MLTC research to better understand disparities. The limited evidence may potentially suggest that, depending on the relational context, MLTC outcomes can vary considerably at the intersections of sociodemographic factors. However, it is too early to draw a definite conclusion from a very small body of non-longitudinal evidence…Future research should prioritise studying MLTC trajectory with an intersectionality lens using longitudinal data (e.g. large-scale electronic health records or individual-level panel surveys that ensure a diverse population) to examine how intersecting disadvantages accumulate onto outcome disparities over time. Moreover, MLTC-intersectionality longitudinal study models should identity and include solution-linked variables to inform more context-responsive MLTC interventions.

**8. Writing Style and Structure: Overall, the manuscript is well written, but some sections are overly dense and could be streamlined to improve readability. There are some redundancies between tables and narrative descriptions. Consider trimming repetitive text.**

- Agreed; performed major streamlining across the whole manuscript.

Journal Requirements

**1. Please ensure that your manuscript meets PLOS ONE's style requirements, including those for file naming.**

- Formatted the title page accordingly, e.g. UK > United Kingdom and NIHR > National Institute for Health and Care Research, per ‘no abbreviation’ rule
- Headings are fixed to level 1 (pt 18, bold), level 2 (pt 16, bold), level 3 (pt 14, bold)
- In-line Vancouver-style citations are now in square brackets, e.g. *“There are modifiable and non-modifiable MLTC risk factors [5, 9]. Individual lifestyle and behavioural attributes [9, 10] are considered modifiable factors and intervention targets, such as stress, sleep, physical activity, smoking, or diet [11].”*
- Changed file names to: “Fig1.tif”, “S1_Table.docx”, “S2_Table.docx”, “S3_Appendix.xlsx”

**2. Please remove any funding-related text from the manuscript and let us know how you would like to update your Funding Statement.**

- Removed all “Funding” related texts from the manuscript as advised
- Indicated on the rebuttal letter that we request PLoS One change the Funding Statement section of the online submission form on our behalf, from current “The author(s) received no specific funding for this work”, to…
  - *The Leicester Real World Evidence Unit is supported by the National Institute for Health and Care Research (NIHR) Applied Research Collaboration East Midlands (ARC EM) and the NIHR Leicester Biomedical Research Centre (BRC).*
  - *KK is supported by the National Institute for Health Research (NIHR) Applied Research Collaboration East Midlands (ARC EM), NIHR Global Research Centre for Multiple Long Term Conditions, NIHR Cross NIHR Collaboration for Multiple Long Term Conditions, NIHR Leicester Biomedical Research Centre (BRC) and the British Heart Foundation (BHF) Centre of Excellence.*

**3. Please include captions for your Supporting Information files at the end of your manuscript, and update any in-text citations to match accordingly.**

- Changed the file names to, S1 Table, S2 Table, and S3 Appendix; and changed in-text citations to match accordingly:
  - “S1 Table for the PRISMA 2020 checklist”
  - “The final risk of bias assessment…details reported in S2 Table.”
  - “The full research results are included as supplementary information (S3 Appendix).”

**4. [added on 17 September 2025] …”thank you for specifying that you do not have personally identifiable data. Could I request you to kindly add your response to data on the comment section or in the cover letter.” (Paula Katrina Maderazo, via email, 16 September 2025)**

- This manuscript is a systematic review which did not make any use of personally identifiable data on human research participants.
  - The Supporting Information file < S3_Appendix_03Sep2025.xlsx > contains search results of a systematic literature review. The key values are: from which database each journal article came, PMID [PubMed IDentifier - unique article reference number], Journal article title, DOI [Digital Object Identifier for journal articles], and what language each article is in.

**References**

1.	Crenshaw KW, editor Demarginalising the intersection of race and gender: a black feminist critique of antidiscrimination doctrine, feminist theory and antiracist politics. University of Chicago legal forum; 1989.

2.	Crenshaw K. Mapping the Margins: Intersectionality, Identity Politics, and Violence against Women of Color. Stanford Law Review. 1991;43(6):1241-99.

3.	Bowleg L. When Black + Lesbian + Woman ≠ Black Lesbian Woman: The Methodological Challenges of Qualitative and Quantitative Intersectionality Research. Sex Roles. 2008;59(5):312-25.

4.	Bowleg L. Evolving Intersectionality Within Public Health: From Analysis to Action. American journal of public health. 2020;111(1):88-90.

5.	Bauer GR. Incorporating intersectionality theory into population health research methodology: Challenges and the potential to advance health equity. Social Science & Medicine. 2014;110:10-7.

6.	Bauer G, Scheim A. Incorporating intersectionality into quantitative research methods in public health. European Journal of Public Health. 2020;30(Supplement_5):ckaa165.744.

7.	Bauer G, Churchill S, Mahendran M, Walwyn C, Lizotte D, Villa-Rueda A. Intersectionality in quantitative research: A systematic review of its emergence and applications of theory and methods. SSM - population health. 2021;14:100798.

8.	Braveman P, Gottlieb L. The social determinants of health: it's time to consider the causes of the causes. Public health reports (Washington, DC : 1974). 2014;129 Suppl 2(Suppl 2):19-31.

9.	Frank J, Abel T, Campostrini S, Cook S, Lin VK, McQueen DV. The Social Determinants of Health: Time to Re-Think? International journal of environmental research and public health. 2020;17(16).

10.	Moreno-Juste A, Gimeno-Miguel A, Poblador-Plou B, Calderón-Larrañaga A, Cano Del Pozo M, Forjaz MJ, et al. Multimorbidity, social determinants and intersectionality in chronic patients. Results from the EpiChron Cohort. Journal of global health. 2023;13:04014.

11.	Moreno-Juste A, Laguna-Berna C, Poblador-Plou B, Calderón-Larrañaga A, Librero J, Lozano-Hernández C, et al. Intersectional analysis of social determinants of health and their association with mortality in patients with multimorbidity. Journal of global health. 2024;14:04229.

12.	Rocca WA, Gazzuola-Rocca L, Smith CY, Grossardt BR, Faubion SS, Shuster LT, et al. Accelerated Accumulation of Multimorbidity After Bilateral Oophorectomy: A Population-Based Cohort Study. Mayo Clinic proceedings. 2016;91(11):1577-89.

13.	Xu X, Mishra GD, Dobson AJ, Jones M. Progression of diabetes, heart disease, and stroke multimorbidity in middle-aged women: A 20-year cohort study. PLoS Med. 2018;15(3):e1002516.

14.	Ashworth M, Durbaba S, Whitney D, Crompton J, Wright M, Dodhia H. Journey to multimorbidity: longitudinal analysis exploring cardiovascular risk factors and sociodemographic determinants in an urban setting. BMJ open. 2019;9(12):e031649.

15.	Kudesia P, Salimarouny B, Stanley M, Fortin M, Stewart M, Terry A, et al. The incidence of multimorbidity and patterns in accumulation of chronic conditions: A systematic review. Journal of multimorbidity and comorbidity. 2021;11:26335565211032880.

16.	Chen S, Marshall T, Jackson C, Cooper J, Crowe F, Nirantharakumar K, et al. Sociodemographic characteristics and longitudinal progression of multimorbidity: A multistate modelling analysis of a large primary care records dataset in England. PLOS Medicine. 2023;20(11):e1004310.

17.	Owen RK, Lyons J, Akbari A, Guthrie B, Agrawal U, Alexander DC, et al. Effect on life expectancy of temporal sequence in a multimorbidity cluster of psychosis, diabetes, and congestive heart failure among 1.7 million individuals in Wales with 20-year follow-up: a retrospective cohort study using linked data. The Lancet Public Health. 2023;8(7):e535-e45.

18.	Harari L, Lee C. Intersectionality in quantitative health disparities research: A systematic review of challenges and limitations in empirical studies. Social science & medicine (1982). 2021;277:113876.

19.	Guan A, Thomas M, Vittinghoff E, Bowleg L, Mangurian C, Wesson P. An investigation of quantitative methods for assessing intersectionality in health research: A systematic review. SSM - population health. 2021;16:100977.

20.	Foong HF, Hamid TA, Ibrahim R, Haron SA. The intersectional effects of ethnicity/race and poverty on health among community-dwelling older adults within multi-ethnic Asian populace: a population-based study. BMC geriatrics. 2021;21(1):516.

21.	Fleitas Alfonzo L, King T, You E, Contreras-Suarez D, Zulkelfi S, Singh A. Theoretical explanations for socioeconomic inequalities in multimorbidity: a scoping review. BMJ open. 2022;12(2):e055264.

22.	Lam A, Keenan K, Myrskylä M, Kulu H. Multimorbid life expectancy across race, socio-economic status, and sex in South Africa. Population studies. 2024:1-26.

23.	Wagner C, Carmeli C, Chiolero A, Cullati S. Life course socioeconomic conditions and multimorbidity in old age - A scoping review. Ageing research reviews. 2022;78:101630.

24.	Tan MMC, Barbosa MG, Pinho P, Assefa E, Keinert AÁ M, Hanlon C, et al. Determinants of multimorbidity in low- and middle-income countries: A systematic review of longitudinal studies and discovery of evidence gaps. Obesity reviews : an official journal of the International Association for the Study of Obesity. 2024;25(2):e13661.

25.	Zandam H, Akobirshoev I, Mitra M. Multiple Chronic Condition Emergency Department Visits Among U.S. Adults: Disparities at the Intersection of Intellectual and Developmental Disabilities Status and Race or Ethnicity. Health equity. 2024;8(1):198-205.

26.	Head A, Fleming K, Kypridemos C, Schofield P, Pearson-Stuttard J, O'Flaherty M. Inequalities in incident and prevalent multimorbidity in England, 2004&#x2013;19: a population-based, descriptive study. The Lancet Healthy Longevity. 2021;2(8):e489-e97.

27.	Ben Hassen C, Fayosse A, Landré B, Raggi M, Bloomberg M, Sabia S, et al. Association between age at onset of multimorbidity and incidence of dementia: 30 year follow-up in Whitehall II prospective cohort study. BMJ. 2022;376:e068005.

28.	Khunti K, Chudasama YV, Gregg EW, Kamkuemah M, Misra S, Suls J, et al. Diabetes and Multiple Long-term Conditions: A Review of Our Current Global Health Challenge. Diabetes Care. 2023;46(12):2092-101.

29.	Bowling CB, Faldowski RA, Sloane R, Pieper C, Brown TH, Dooley EE, et al. Multimorbidity trajectories in early adulthood and middle age: Findings from the CARDIA prospective cohort study. Journal of multimorbidity and comorbidity. 2024;14:26335565241242277.

30.	Bisquera A, Turner EB, Ledwaba-Chapman L, Dunbar-Rees R, Hafezparast N, Gulliford M, et al. Inequalities in developing multimorbidity over time: A population-based cohort study from an urban, multi-ethnic borough in the United Kingdom. The Lancet Regional Health – Europe. 2022;12.

31.	Coles B, Zaccardi F, Ling S, Davies MJ, Samani NJ, Khunti K. Cardiovascular events and mortality in people with and without type 2 diabetes: An observational study in a contemporary multi-ethnic population. Journal of Diabetes Investigation. 2021;12(7):1175-82.

32.	O’Campo P, Dunn JR. Rethinking social epidemiology: towards a science of change: Springer Science & Business Media; 2012.

33.	Mena E, Bolte G. Intersectionality-based quantitative health research and sex/gender sensitivity: a scoping review. International journal for equity in health. 2019;18(1):199.

34.	di Lego V, Lazarevič P, Luy M. The male-female health-mortality paradox. Encyclopedia of gerontology and population aging: Springer; 2022. p. 5070-7.
